# The Safety, Clinical, and Neurophysiological Effects of Intranasal Ketamine in Patients Who Do Not Respond to Electroconvulsive Therapy: Protocol for a Pilot, Open-Label Clinical Trial

**DOI:** 10.2196/30163

**Published:** 2022-01-17

**Authors:** Yuliya Knyahnytska, Reza Zomorrodi, Tyler Kaster, Daphne Voineskos, Alisson Trevizol, Daniel Blumberger

**Affiliations:** 1 Centre for Addiction and Mental Health Toronto, ON Canada

**Keywords:** intranasal, racemic ketamine, NMDA antagonist, treatment resistant depression, electroconvulsive therapy nonresponders, drug, treatment, ketamine, depression, mental health, safety, neurophysiological, side effect, biomarker, clinical trial, alternative

## Abstract

**Background:**

Major depressive disorder is among the most disabling illnesses worldwide, with a lifetime prevalence of 16.2%. Research suggests that 20% to 40% of patients with depression do not respond to pharmacotherapy, developing treatment-resistant depression. Electroconvulsive therapy is the gold standard for treating individuals with treatment-resistant depression, with remission rates of approximately 75% to 90%. However, 10% to 25% of patients do not respond to electroconvulsive therapy, and many are unable to tolerate it due to the side effects. Both groups are considered to be patients who do not respond to electroconvulsive therapy, because both groups continue to exhibit symptoms of severe depression, have a limited number of treatment options available, and are in need of rapid treatment. Ketamine, an N-methyl-D-aspartate receptor antagonist, has been shown to exert rapid antidepressant effects in patients with treatment-resistant depression when administered in subanesthetic doses through 40-minute intravenous infusions. Recently, a ketamine compound, esketamine (Spravato), that is administered through the intranasal route received regulatory approval by the US Food and Drug Administration and Health Canada to treat depression. However, esketamine is challenging to access due to high costs and limited availability. Racemic ketamine (rketamine) is cheap and easy to access; however, the effects in patients who have not responded to electroconvulsive therapy have yet to be understood or tested. This study will use transcranial magnetic stimulation to study mechanisms of human brain cortical physiology at the systemic level to identify neurobiomarkers of response.

**Objective:**

The objective of this open-label pilot clinical trial is to test the feasibility and safety of intranasal ketamine in patients who have not responded to electroconvulsive therapy. The primary outcome is to determine the feasibility of a larger randomized controlled trial to test the efficacy of intranasal ketamine for patients who have not responded to electroconvulsive therapy for clinical indicators in unipolar depression. The secondary outcome is to determine the preliminary effects of an intervention on clinical outcomes, such as depressive symptoms, suicidal ideation, and quality of living. The third outcome is to explore neurophysiological changes as measured by transcranial magnetic stimulation electromyography and electroencephalography to measure changes in cortical excitability as potential predictors of clinical response.

**Methods:**

A sterile solution of racemic ketamine hydrochloride will be administered twice per week for 4 weeks (8 sessions) intranasally to patients with treatment-resistant depression who did not respond to or could not tolerate an acute course of electroconvulsive therapy. We will recruit 25 adults (24-65 years old) over the course of 2 years from an academic psychiatric hospital in Toronto, Canada.

**Results:**

This study has received ethics approval, and funding has been secured. The study is currently active.

**Conclusions:**

This is the first study to test repeated doses of intranasal rketamine in patients who have not responded to electroconvulsive therapy for depression. Results from this study will (1) inform the development of a larger adequately powered randomized controlled trial to test the efficacy of intranasal ketamine for depression and (2) determine potential neurophysiological markers of clinical response.

**Trial Registration:**

Clinical Trials.gov NCT05137938; http://clinicaltrials.gov/ct2/show/NCT05137938

**International Registered Report Identifier (IRRID):**

PRR1-10.2196/30163

## Introduction

Depressive disorders remain common, severe, and debilitating, with a lifetime prevalence estimated at 16.2% [1]. Research suggests that between 20% and 40% of patients with depression do not respond adequately to pharmacotherapies such as selective serotonin and serotonin–norepinephrine reuptake inhibitors, tricyclic antidepressants, or other psychotherapeutic interventions [2], and thus, are diagnosed with treatment-resistant depression [3]. Electroconvulsive therapy continues to be the gold standard therapeutic approach for treatment-resistant depression [3]. The Consortium for Research in Electroconvulsive Therapy [4] reports a 65% remission rate after 10 sessions that is consistent across the spectrum for bipolar and unipolar depression [5], with some previous studies also reporting remission rates as high as 70% to 90% [4,6]. However, despite these high efficacy rates, 10% to 30% of patients do not respond to electroconvulsive therapy, and these patients are left with very few treatment options [7].

Ketamine, a noncompetitive high-affinity *N*-methyl-D-aspartate (NMDA) receptor antagonist, has been shown to exert rapid and robust antidepressant effects in patients with treatment-resistant depression [8]. Studies have demonstrated that a single intravenous infusion of an NMDA antagonist resulted in significant decreases in depressive symptoms [9], making ketamine an attractive treatment option with the ability to simultaneously reduce neuronal activity in the limbic and subcortical regions while increasing activity in the prefrontal cortex [10], an area of core dysfunction reported in those with depression [10]. Numerous studies [11,12] have demonstrated that a subanesthetic dose of ketamine (0.5-0.8 mg/kg) over 40-minute intravenous infusion produces rapid antidepressant effects in patients with treatment-resistant depression. It has also been reported that the route of administration of ketamine can influence the clinical antidepressant effects of treatment owing to its extensive first-pass metabolism; specifically, the highest bioavailability of ketamine is achieved via intravenous infusion, while oral administration yields the lowest bioavailability [13]. Findings suggest that 70% of patients with treatment-resistant depression respond to 1 to 3 administrations of ketamine, and 30% to 60% of patients with treatment-resistant depression experience remission of their depressive symptoms. Research suggests that most responses occur if ketamine is administered in 6 or more sessions over a >2-week period [8,12].

Although intravenous ketamine has demonstrated rapid antidepressant effects, its delivery method remains challenging because it requires specialized expertise and equipment for administration [11]. Intranasal drug delivery, while preserving the rapid onset of therapeutic action, offers a route to the brain that bypasses problems associated with gastrointestinal absorption, first-pass metabolism, and the blood-brain barrier, and thus, minimizes the inconvenience and discomfort of parenteral administration [14]. Studies [15] indicate that intranasal rketamine has an absolute bioavailability of 50%, with a maximum plasma concentration achieved at approximately 20 minutes. This is consistent with other studies [13,15-17] in which the reported bioavailability of intranasal rketamine was demonstrated to be 45% to 50%. Converging evidence indicates that using intranasal ketamine with a dose range of 50-175 mg taken in intervals of 3 to 7 days can positively affect mood symptoms with a limited number of side effects [10]. Recently, esketamine (Spravato) received regulatory approval from the US Food and Drug Administration and Health Canada. However, it remains out of reach for most people with depression due to high costs and limited availability. To our knowledge, the clinical effects of intranasal rketamine have not yet been studied in a group of individuals with treatment-resistant depression who have not responded to electroconvulsive therapy.

Noninvasive brain stimulation neurophysiological tools, such as transcranial magnetic stimulation (TMS), offer an elegant opportunity to study mechanisms of human cortical physiology at the systemic level. The combination of TMS with a central nervous system pharmacological agent, such as ketamine, provides a platform to explore the neurophysiological impact of ketamine and allows neurophysiological biomarkers of treatment response to be identified. Until recently, only a few studies [16-18] have attempted to examine excitatory and inhibitory circuits by using a range of TMS protocols during infusion of incremental doses of ketamine, primarily using very low or single doses of ketamine in healthy volunteers. Currently, the research on predicting therapeutic response in those with mood disorders, measured by changes in cortical excitability, continues to be in very early stages and has not been systematically tested, making this proposal uniquely positioned among other neurophysiological studies [16,17,19] on ketamine and TMS. Our group has previously demonstrated impaired γ-aminobutyric acid inhibition from the motor cortex in patients with depression, a finding that was most pronounced in patients with treatment-resistant depression [18]. Additionally, Croarkin et al [20] demonstrated impaired NMDA receptor-mediated excitation in adolescent depression. The impact of ketamine on an excitatory or inhibitory cortical network will be assessed using neurophysiological tools such as TMS, specifically via short-interval cortical inhibition paradigms [21],

In this study, we aim to assess the safety and feasibility of intranasal rketamine in patients with unipolar depression who did not respond to the acute course of electroconvulsive therapy to inform a larger randomized controlled trial and examine potential neurophysiological biomarkers of response. TMS- electromyography (EMG) and electroencephalography (EEG) paradigms can be used to investigate the impact of ketamine on cortical activities via intracortical facilitation and short-interval cortical inhibition paradigms [19,22-26]. Based on accumulating evidence supporting the efficacy and tolerability of ketamine, we hypothesize that intranasal rketamine administered twice per week over 4 weeks will (1) result in improvement in depressive; (2) be safe and well tolerated in patients with treatment-resistant depression who could not tolerate or have not responded to electroconvulsive therapy; (3) result in neurophysiological changes. The results of this study will provide an important characterization of the neurophysiological effects of rketamine on cortical neurophysiology [19,22-26], which may serve as a ketamine biomarker and would be a crucial breakthrough in determining potential predictors of clinical response for depression.

## Methods

### Design

This is an open-label pilot study to assess the feasibility of conducting a randomized controlled trial to test the safety, tolerability, and efficacy of intranasal ketamine in patients with treatment-resistant depression who did not respond or were not able to tolerate a course of acute electroconvulsive therapy.

### Ethics Approval

This study has received Centre for Addiction and Mental Health (CAMH) research ethics board (095-2019) and Health Canada approval. For this pilot trial, all relevant adverse events and all serious adverse events will be reported if they meet applicable reporting requirements. All data monitoring, auditing, and harms reporting will be performed according to CAMH research ethics board and regulatory standards.

### Recruitment and Feasibility

#### Recruitment

Over a period 2 years, we intend to recruit 25 adults aged 24 to 65 years old diagnosed with treatment-resistant depression from one site (CAMH).

#### Participants

Patients will be assessed for eligibility based on inclusion and exclusion criteria.

#### Inclusion and Exclusion Criteria

Inclusion criteria are patients who (1) have a DSM-5 diagnosis of nonpsychotic major depressive disorder, confirmed by the Mini-International Neuropsychiatric Interview; (2) meet criteria for being nonresponsive to electroconvulsive therapy in the current episode (nonresponse is defined as lack of improvement in depressive symptoms after 8 acute sessions of electroconvulsive therapy, confirmed with the Hamilton Rating Scale for Depression (HRSD-24 score >14), and nontolerability is determined by a brain stimulation psychiatrist based on side effects, such as postictal confusion, significant cognitive impairment, severe worsening in anxiety preventing a patient from continuous treatment, or failure to secure intravenous access safely); (3) exhibit moderate to severe symptoms of depression (HRSD-24 score >14); (4) are capable of providing consent; (5) are outpatients; (6) are able to speak and understand English; and (7) are aged 24 to 65 years, inclusive.

Exclusion criteria are patients (1) with a history of a substance use disorder within the past month or lifetime history of ketamine substance use disorder, confirmed by the Mini-International Neuropsychiatric Interview; (2) with concomitant major unstable medical illness (eg, poorly controlled blood pressure; enlarged prostate; unresolved urinary related issues); (3)with a confirmed pregnancy or the intention to become pregnant and breastfeeding during the study (self-report), and female participants of reproductive age must be willing to use a medically acceptable method of birth control that includes highly effective (eg, approved hormonal contraceptives, intrauterine device, tubal ligation), double barrier (eg, male condom with a diaphragm, male condom with cervical cap) methods of contraception, or abstinence if that is the usual and preferred lifestyle of the participant; (4) with cardiac decompensation or heart failure; (5) with a DSM-5 diagnosis of any primary psychotic disorder, bipolar disorder, obsessive-compulsive disorder, or current posttraumatic stress disorder, confirmed by the Mini-International Neuropsychiatric Interview; (6) with a diagnosis of severe personality disorder, assessed during the initial consultation with a physician at the study site prior to study entry; (7) with any significant neurological disorder (eg, a space-occupying brain lesion, a history of stroke, a cerebral aneurysm, a seizure disorder, Parkinson disease, Huntington chorea, multiple sclerosis), assessed through medical history review during the initial consultation with a physician at the study site prior to study entry; (8) with a medical condition, taking medication, or with a laboratory abnormality that could cause a major depressive episode or significant cognitive impairment in the opinion of the investigator; (9) requiring a benzodiazepine with a dose equivalent to lorazepam 2 mg/day or higher; (10) on any anticonvulsant (eg, lamotrigine) or opioid medication due to the potential of these medications to limit the efficacy of ketamine; (11) with the inability to communicate in spoken and written English fluently enough to complete the required study assessments due to a language barrier or a noncorrectable clinically significant sensory impairment (ie, cannot hear or see well enough to complete clinical assessments); (12) with any cognitive or physical impairment which may potentially interfere with intranasal ketamine administration or the patient’s ability to stay in the same place for a 2-hour monitoring supervision, assessed through medical history review during the initial consultation with a physician at the at the study site prior to study entry; (13) with any intracranial implants (eg, aneurysm clips, shunts, cochlear implants) or any other metal object within or near the head, excluding the mouth, that cannot be safely removed given that we will be using TMS-EMG/EEG; (14) with the inability to secure escort to accompany them back home after ketamine sessions; or (15) with any known allergy to ketamine or any component or ingredient of the ketamine preparation.

#### Discontinuation Criteria

Participants will be discontinued from the study if they experience clinically significant worsening depression symptoms (50% increase in HDRS-24 scores from baseline on 2 consecutive ratings); require in-patient hospitalization due to the presence of clinically significant suicidal ideation with imminent intent or attempted suicide; develop clinically significant worsening of mood, psychotic, or physical symptoms (assessed by a study physician); miss more than 2 consecutive treatments during the study; develop any medical illness that may be unstable; experience a seizure; become pregnant; or withdraw consent.

#### Feasibility

We believe recruiting 25 participants over the course of 2 years is feasible. Participants will be recruited through the Temerty Centre for Brain Intervention at CAMH, which treats approximately 150 to 200 patients with depression with electroconvulsive therapy per year. The principal investigator is a staff psychiatrist at the Mood and Anxiety Division, which provides outpatient services for a large population with mood disorders at local and provincial levels. In addition, this protocol has been piloted with 3 patients who were nonresponsive to electroconvulsive therapy, and the patients were able to comply with a protocol of 2 sessions per week for 8 treatment sessions given very close clinical supervision in a trial (unpublished data, Y. Knyahnytska).

#### Consent to Participate

Informed consent will be obtained from each individual who agrees to participate in the trial prior to and throughout participation. Patients meeting criteria will be referred to the study coordinator to discuss the study purpose, procedures, potential risks, and rights as research participants. Consent forms describing the study intervention, study procedures, and risks will be given to each participant, and written documentation of informed consent will be required prior to completing the initial screening visit and starting the study intervention. Once consent is obtained, the research personnel will confirm that inclusion and exclusion criteria are met before proceeding with baseline testing. Patients will be informed that they can withdraw from the study at any point. A copy of the information and consent documents will be given to the participants for their records. The informed consent process will be documented, and the form signed before the participant undergoes any study-specific procedures. The rights and welfare of the participants will be protected by emphasizing to them that the quality of their medical care will not be adversely affected if they decline to participate in this study.

Data confidentiality and confidentiality of the identities of the individuals participating in this study will be strictly maintained. Data forms that include identifying information will be kept in locked cabinets. Only the unique ID number assigned by the research coordinator will be used to represent participants during data entry, data transfer, data analysis, or other file management procedures. All information linking their identity will be kept separate from the research records. All information entered into a computer will be stored in a password-protected encrypted file format on a secure server. If any participant withdraws from the study, any research information recorded for or resulting from participation prior to the date that the participant formally withdrew consent will continue to be stored and used (in the manner described above) for research purposes (and will be disclosed by the investigators); however, no new data will be collected. Withdrawing from the study will not have any consequences for the participant. Participants' identities will not be revealed in the publication or presentation of any results from this study.

### Intervention

#### Drug Characteristics, Distribution, and Storing

A sterile form of racemic ketamine hydrochloride will be dispensed through the CAMH pharmacy and will be administered intranasally using an atomizer provided by the pharmacy (MAD300, Teleflex). The CAMH research pharmacy will order the investigational product from the supplier (Sandoz Canada Inc) on behalf of the research team with the study investigator’s authorization and will be responsible for the receipt and responsible destruction of the investigational product. The investigational product will be stored between 15 ºC and 30 ºC, protected from light and heat and discarded within 28 days of initial use.

#### Drug Administration and Scheduling

Intranasal rketamine will be administered twice per week for 4 weeks (8 treatment sessions in total). The dosage schedule will be determined based on participants’ weight, clinical response, and tolerability (Multimedia Appendix 1). Given that the absolute bioavailability of intranasal ketamine is 45% to 50 % compared with that of intravenous administration [13,14], intranasal doses will range from 1 mg/kg to 1.6 mg/kg to represent usual intravenous doses of 0.5-0.8 mg/kg and to account for reduced bioavailability of the intranasal form. Participants will be started at the lowest dose during the first treatment session (average 50 mg of intranasal ketamine administered in 2 syringes with 25 mg per syringe).

#### Dose Adjustment

If the first session is tolerated well, and the patient has no side effects, the session 2 dose will be increased to 1 mg/kg (to represent an intravenous dose of 0.75 mg/kg adjusted to 50% bioavailability). If both sessions are tolerated well, session 3 will start on a full therapeutic dose (1.5-1.6 mg/kg to represent intravenous doses of 0.75-1 mg/kg). Participants will have a weekly visit with the study’s medical doctor, and doses will be monitored and adjusted based on tolerability and clinical response; however, they will not exceed 1.6 mg/kg.

#### Monitoring Schedule

All patients will stay on-site for the administration and 2-hour postintervention monitoring period, per consensus guidelines in ketamine administration [27]. Patients will be provided with a separate quiet space, noise-cancellation headphones, and an eye mask to minimize environmental disruptions. All patients will be observed 1:1 or 1:2 for the entire duration of the treatment session by trained personnel. The medical doctor is present on-site during the session to address any urgent requests and provide support to the team and patient. Vitals will be taken and recorded consistently during every 30 minutes during the 2-hour monitoring period after administration. A medical team will manage any emergencies that arise, and appropriate medications will be provided to manage treatment-related side effects and any adverse events, if needed (defined by clinical site policies and regulations). Clinical and neurophysiological assessments will be administered according to a schedule for study visits by trained research personnel (Multimedia Appendix 2).

#### Potential Risks and Mitigation Strategies

Given the potential risks described below, trained medical personnel will be present during the administration and for the entire duration of the 2-hour monitoring period.

Drug-related risks include (1) psychiatric symptoms such as fatigue, dizziness, anxiety, visual and auditory disturbances, panic attacks, increased irritability, or changes in mood and behavior; (2) medical symptoms such as transient increases in blood pressure and heart rate, an increase in need to urinate, headaches, vision changes, chest pain, shortness of breath, confusion, memory impairment, anaphylaxis; and (3) rare risk of dependency. Ketamine is classified as a schedule I controlled substance due to its potential for abuse and addiction and can be abused in a number of ways, including via injection, snorting, or orally [8]. Ketamine can produce vivid dreams and feel that the mind is separated from the body. Regular users of ketamine can become tolerant to the dissociative effects of the drug, meaning more and more is needed to achieve the same effect [8]. To address these risks, in this trial, ketamine will be dispensed by the research pharmacy and administered by a trained medical professional, and the patient will receive close supervision and monitoring. Doses are individually calculated, and treatment sessions are structured to prevent tolerance building. Patients will be strongly discouraged from using ketamine outside of the context of this trial and will be informed that if they have more questions, they can be discussed with their medical provider or study physician.

A potential risk in clinical assessments is that answering multiple questions can, at times, be distressing. These adverse reactions are primarily brief and transient and rarely have any long-term implications.

The ability of TMS to noninvasively stimulate brain areas presents a significant advance beyond techniques that require the invasive method of direct cortical or transcranial electrical stimulation. Magnetic fields pass through the scalp and skull without the impedance encountered by direct electrical stimulation, permitting enhanced control over the site and intensity of stimulation. In numerous studies [23,24], single-pulse TMS has been found to pose no significant health risk to properly screened healthy volunteers. Single-pulse TMS is now in routine clinical diagnostic use in hundreds of neurophysiological laboratories worldwide, and the induced electrical current is well below that which is expected to cause harm to nervous tissue; thus, stimulation at <1 Hz carries virtually no risk of seizure and is therefore classified as a nonsignificant risk device [23,24].

### Study Measurements

Given that this trial recruits participants who received at least one electroconvulsive therapy session, and therefore, underwent blood work, electrocardiography, and medical clearance by anesthesia services in accordance with standard clinical procedures, we will use these parameters for this trial. The results will be available for screening and review by the study’s medical doctor prior to the start of treatment. Tests completed within the 6 months prior to screening will be used unless new medical symptoms requiring further investigation emerge, in which case, the tests will be repeated. Blood tests will include complete blood count with differential tests (white blood cells, red blood cells, hemoglobin, hematocrit, mean corpuscular volume, mean corpuscular hemoglobin, mean corpuscular hemoglobin concentration, red cell distribution width, mean platelet volume, platelet, nucleated red blood cell count, neutrophil count, lymphocyte count, monocyte count, eosinophil count, basophil count) and blood chemistry tests for liver function (alanine aminotransferase, aspartate aminotransferase), kidney function (blood urea nitrogen, creatinine), electrolyte levels (sodium, potassium, chloride, phosphate), and thyroid function (thyroid-stimulating hormone). Electrocardiography results will be reviewed by the study’s medical doctor. Participant blood pressure will also be monitored before, during, and after treatment.

Clinical assessments will include (1) the Mini-International Neuropsychiatric Interview, to assess current and lifetime depression and other psychiatric disorders and to clarify psychiatric inclusion and exclusion criteria; (2) the 24-item Hamilton Rating Scale for Depression [28], as the primary outcome measure (and a score greater than or equal to 14 will be used to establish eligibility at study entry); (3) the Scale of Suicide Ideation [29], administered at baseline, weekly, at posttreatment, and at follow-up to monitor for any safety concerns and a secondary outcome measure to assess suicidality; and the(4) World Health Organization Disability Assessment Schedule 2.0 [30], used as a standard measure of disability promoted and administered at baseline and posttreatment to assess changes in individual level of functioning.

Demographic information, medical history, concomitant medications, and antidepressant treatment history form information will also be assessed.

Monitoring assessment during treatment sessions will include (1) vital signs collected every 30 minutes during a 2-hour monitoring session (blood pressure, heart rate, and oxygen levels) and (2) behavior changes assessed through observation.

Neurophysiological assessments will include (1) the Transcranial Magnetic Stimulation Adult Safety Screen to assess for potential TMS risk factors; and (2) TMS-EMG/EEG (Multimedia Appendix 2). Our neurophysiological measures have been established and have a high test–retest reliability (ie, intraclass correlation >0.9) [31,32]. Data analysis will be performed using semiautomated methods developed and validated by our group [31,32]. TMS pulses will be administered to the left dorsolateral prefrontal cortex, using a figure-of-eight coil (7 cm), and 2 Magstim 200 stimulators (Magstim Company Ltd) connected via a Bistim module, and motor evoked potential data will be collected using commercially available software (Signal, Cambridge Electronics). Each TMS session will include the establishment of the individual threshold for stimulation motor cortex, and dorsolateral prefrontal cortex localization will be performed according to previously published methods [31,32]. Resting motor threshold will be determined by applying single pulses of TMS to the motor cortex while the coil is placed at the optimal position to elicit motor evoked potentials from the right abductor pollicis brevis muscle. The resting motor threshold is defined as the minimum stimulus intensity that elicits a motor evoked potential of >50 μV in more than 5 of the 10 trials [33,34]. Electromyography will be recorded from the abductor pollicis brevis with Ag–AgCl electrodes placed over the belly of the muscle. The signal will be amplified (Intronix Technologies Corporation Model 2024F), filtered (bandpass 2 Hz-5 kHz), digitized at 5 kHz (Micro 1401, Cambridge Electronics Design), and stored in a laboratory computer for offline analysis. The participants will be instructed to relax throughout the study. Trials contaminated with voluntary muscle activity will be discarded.

EEG will be used to evaluate TMS-induced cortical evoked activity. EEG recordings will be acquired through a 64-channel EEG system [35]. A 64-channel EEG cap will be used to record the cortical signal, and 4 electrodes will be placed on the outer side of each eye and above and below the left eye for eye movement artifacts. All electrodes will be referenced to an electrode placed on the vertex positioned posterior to the Cz electrode. Direct current EEG signals will be recorded with a 20 kHz sampling rate and with a low-pass filter of 300 Hz, which, in pilot experiments [35], was shown to avoid saturation of amplifiers and minimize the TMS-related artifact. The EEG data will be downsampled to 1 kHz and segmented with respect to the TMS stimulus, such that each epoch includes 1000 ms of prestimulus baseline and 1000 ms of poststimulus activity. Epochs will be baseline corrected with respect to the TMS-free prestimulus interval (1000 ms to 110 ms prior to the TMS). The baseline-corrected poststimulus intervals (approximately 25 ms-1000 ms) that are not contaminated by TMS artifact will be extracted and digitally filtered using a zero-phase shift 1-100 Hz bandpass filter (48 dB per octave). Records will be manually reviewed at this stage, and trials contaminated with muscle activity, movement, and TMS artifacts will be excluded from further analysis. Finally, the average signals at each recording site will be computed from the movement-free epochs (approximately 80 trials per participant) and fed into an automated eye-blink correction algorithm [36]. The eye-blink corrected average EEG waveforms will be imported into MATLAB (The MathWorks Inc), and further analyses will be carried out utilizing the EEGLAB toolbox [37-39]. Further methods in this approach will be conducted according to previously published combined TMS/EEG studies [35].

### Statistical Analysis

#### Statistical and Analytical Plans

Baseline participant characteristics will be reported and described using summary statistics—mean and standard deviation for continuous data and number and proportion for categorical data. The primary analysis to determine if there is a statistically significant effect of intranasal ketamine on depressive symptoms will be the paired 1-tailed *t* test of the HDRS-24 score at baseline to week 4. We will also report the standardized mean difference as a measure of effect size (small: 0.2; medium: 0.5; large: 0.8 [40]). For tolerability and safety outcomes, we will report the number and proportion of individuals who experience a transient increase in blood pressure, agitation, and behavioral disturbance. To assess suicidality change, we will use the 1-tailed paired *t* test from baseline to week 4 of the Scale of Suicide Ideation score and calculate the standardized mean difference. For neurophysiology, we will use the 1-tailed independent *t* test to compare components of TMS-evoked responses at left dorsolateral prefrontal cortex. In addition, we will use the nonparametric cluster-based permutation test to investigate any significant changes in overall EEG channels.

#### Sample Size

CAMH treats approximately 250 new patients with depression each year with electroconvulsive therapy. Assuming 25% to 30% nonresponse or intolerability and that, of these individuals, 20% will be eligible for treatment, we expect to be able to recruit 25 participants over the course of 24 months.

## Results

This study has received ethics approval. We have started recruitment for the trial and anticipate having initial results in spring 2022.

## Discussion

To our knowledge, this is the first study to test repeated doses of rketamine delivered intranasally in patients with major depressive disorder who did not respond (either clinically or due to their inability to tolerate) to an acute course of electroconvulsive therapy. The use of neurophysiological tools to assess changes in cortical excitability will provide preliminary data for potential biomarkers of response, which can be further assessed in a larger clinical trial. A lack of control group and small sample size are limitations of the current protocol. However, given that it is a pilot open-label clinical trial, the sample size is sufficient (ie, the goal is not to assess generalizability or statistical significance). The lack of blinding in the control group is a common concern in ketamine trials [27], because a placebo with comparable effects does not exist, and it is impossible to ensure blinding to the full extent. Because the study is being conducted at a single site of a large psychiatric academic facility, the results may not be transferable to a broader population.

Results will be presented during scientific conferences, in clinical rounds, and publications in relevant journals.

## Appendix

Multimedia Appendix 1 Dosage schedule for intranasal rketamine.

Multimedia Appendix 2 Study visits.

## Abbreviations

CAMH: Centre for Addiction and Mental Health
DSM-5: Diagnostic and Statistical Manual of Mental Disorders, 5th edition
EMG: electromyography
EEG: electroencephalography
GABA: γ-aminobutyric acid
NMDA: N-methyl-D-aspartate
rketamine: racemic ketamine
TMS: transcranial magnetic stimulation
