# Supplementary material for: The Safety, Clinical, and Neurophysiological Effects of Intranasal Ketamine in Patients Who Do Not Respond to Electroconvulsive Therapy: Protocol for a Pilot, Open-Label Clinical Trial
Source: JMIR Res Protoc. 2022 Jan 17;11(1):e30163. doi: 10.2196/30163 (PMC8804953; doi:10.2196/30163)
Supplement: Multimedia Appendix 1 [file resprot_v11i1e30163_app1.docx]

**Appendix 1: Table 1. Intranasal (*IN)* _r_ketamine proposed dosing schedule**

| **Week** | **Session** | **Proposed dose*** |
| --- | --- | --- |
| 1 | 1 | Start-up dose to ensure tolerability. 50 mg of ketamine will be administered in increments of 25 mg into each nostril. |
|  | 2* | If previous dose tolerated and no side effects, 0.75 mg/kg |
| 2 | 3* | Full dose* (1 mg/kg) |
|  | 4* | Full dose* |
| 3 | 5* | Full/or adjusted further dose* |
|  | 6* | Full/or adjusted further dose* |
| 4 | 7* | Full/or adjusted further dose* |
|  | 8* | Full/or adjusted further dose* |
| *Dose will vary depending on clinical response and tolerability. Doses will be adjusted during the whole duration of the trial if needed | | |
