# Supplementary material for: The Safety, Clinical, and Neurophysiological Effects of Intranasal Ketamine in Patients Who Do Not Respond to Electroconvulsive Therapy: Protocol for a Pilot, Open-Label Clinical Trial
Source: JMIR Res Protoc. 2022 Jan 17;11(1):e30163. doi: 10.2196/30163 (PMC8804953; doi:10.2196/30163)
Supplement: Multimedia Appendix 2 [file resprot_v11i1e30163_app2.docx]

**Appendix 2: Table 2. Study visits**

| **Visits** | **Procedures** | **Duration** |
| --- | --- | --- |
| Visit 1  (Screening/Baseline Assessment) | - Consent - MINI - ATHF - Demographic and Medical Information Form - SSI - HDRS-24 - TASS - WHODAS 2.0 - TMS-EMG, EEG (pre-treatment) | ~30 min  ~30 min  ~10 min  ~5 min  ~10 min  ~30 min  ~5 min  ~5 min  ~60 min |
| Visit 2 (treatment session #1) within 7 days post-baseline assessment | - Treatment Session/Monitoring - TMS-EMG, EEG (post-treatment) | ~120 min  ~60 min |
| Visit 3 (treatment session #2) | - Treatment Session/Monitoring - SSI - HDRS-24 | ~120 min  ~10 min  ~30 min |
| Visit 5 (treatment session #3) | - Treatment Session/Monitoring | ~120 min |
| Visit 6 (treatment session #4) | - Treatment Session/Monitoring - SSI - HDRS-24 | ~120 min  ~10 min  ~30 min |
| Visit 7 (treatment session #5) | - Treatment Session/Monitoring | ~120 min |
| Visit 8 (treatment session #6) | - Treatment Session/Monitoring - SSI - HDRS-24 | ~120 min  ~10 min  ~30 min |
| Visit 9 (treatment session #7) | - Treatment Session/Monitoring | ~120 min |
| Visit 10 (treatment session #8) | - Treatment Session/Monitoring | ~120 min |
| Visit 11 (Post-Treatment) within 7 days after last ketamine session | - SSI - HDRS-24 - WHODAS 2.0 - TMS-EMG, EEG - MD follow up | ~10 min  ~30 min  ~5 min  ~60 min |
| Visit 12-Final Follow up assessment (1-month) | - SSI - HDRS-24 | ~10 min  ~30 min |
